# A Blockchain-Based Multi-Mobile Code-Driven Trust Mechanism for Detecting Internal Attacks in Internet of Things

**DOI:** 10.3390/s21010023

**Published:** 2020-12-22

**Authors:** Noshina Tariq, Muhammad Asim, Farrukh Aslam Khan, Thar Baker, Umair Khalid, Abdelouahid Derhab

**Affiliations:** 1 Department of Computer Science, National University of Computer and Emerging Sciences, Islamabad 44000, Pakistan; i131502@nu.edu.pk (N.T.); muhammad.asim@nu.edu.pk (M.A.); i171016@nu.edu.pk (U.K.); 2 Center of Excellence in Information Assurance (CoEIA), King Saud University, Riyadh 11653, Saudi Arabia; abderhab@ksu.edu.sa; 3 Department of Computer Science, University of Sharjah, Sharjah 27272, UAE; tshamsa@sharjah.ac.ae

**Keywords:** trust, energy-efficiency, blockchain, multi-mobile code, internet of things

## Abstract

A multitude of smart things and wirelessly connected Sensor Nodes (SNs) have pervasively facilitated the use of smart applications in every domain of life. Along with the bounties of smart things and applications, there are hazards of external and internal attacks. Unfortunately, mitigating internal attacks is quite challenging, where network lifespan (w.r.t. energy consumption at node level), latency, and scalability are the three main factors that influence the efficacy of security measures. Furthermore, most of the security measures provide centralized solutions, ignoring the decentralized nature of SN-powered Internet of Things (IoT) deployments. This paper presents an energy-efficient decentralized trust mechanism using a blockchain-based multi-mobile code-driven solution for detecting internal attacks in sensor node-powered IoT. The results validate the better performance of the proposed solution over existing solutions with 43.94% and 2.67% less message overhead in blackhole and greyhole attack scenarios, respectively. Similarly, the malicious node detection time is reduced by 20.35% and 11.35% in both blackhole and greyhole attacks. Both of these factors play a vital role in improving network lifetime.

## 1. Introduction

Internet-of-Things (IoT) is about anytime, anywhere service provisioning to end-users; thanks to a plethora of static and mobile devices, such as actuators, sensors, and controllers [1]. It reinforces quality and reliability in enterprise systems by providing promising processing solutions and resource management [2]. The intense communications between all these devices, some of which are smart (i.e., embedded with cognitive capabilities), have allowed IoT to penetrate every domain from home automation to industry 4.0 revolution [3,4]. In this view, various technologies, such as fog, Wireless Sensor Networks (WSNs), 5G, etc., play an important role in augmenting massive and smart interconnection notion [5,6]. For instance, WSNs, consisting of resource-constrained smart entities known as Sensor Nodes (SNs), provide a foundation for IoT to collect data. WSNs are developed using different technologies, such as MicroElectroMechanical Systems (MEMS), System on Chip (SoC), wireless communication, and low-powered embedded technologies [7]. Currently, it has diverse areas of applications, like military, smart transportation, and many more [8]. However, some critical challenges need to be encountered, like network lifespan [9], decentralization, transparency, risks of data interoperability, network privacy, and security vulnerabilities [10,11]. According to Gartner estimation, approximately twenty-five billion physical entities will be interconnected through IoT to operate as a single network by 2021 [12]. Since IoT generates massive and highly sensitive data, needs for proficient capturing, transmission, processing, analysis, sharing, and protection against misuses and/or attacks cannot be ignored [13].

The large volume of data exercises much pressure on the IoT infrastructure, raising concerns such as network congestion and high latency [14]. It would undermine some IoT-based applications, such as real-time traffic control in a smart city. Apart from traffic congestion and latency, energy conservation and scalability are among the most concerning aspects of such applications. Conventional security mechanisms do not consider restrained energy at the node level, and hence deplete SNs‘energy that threatens the network lifespan [15]. On the other hand, centralized security mechanisms do not cater to the myriad of connected IoT devices due to poor scalability [16]. In such scenarios, the delays go even higher. Moreover, centralized security options, such as centralized trust assessment mechanisms, also go against the spirit of distributively deployed IoT. They are highly prone to single-point-of-failure and scalability issues. Thus, it is important to mitigate energy consumption and centralized (i.e., many-to-one) traffic flow to achieve optimum network lifespan, scalability, and robustness. It also highlights the need for decentralized security solutions. It can help in improving network lifespan and eliminating single-point-of-failure, network congestion, and delay issue. By nature, most IoT devices are resource-constrained in terms of limited computation, storage, and communication. Capacities to perform complex security operations to protect IoT devices require many resources, which these devices do not have. Thus, blockchain has been considered as a powerful technology to encounter such bottlenecks within WSNs and 5G networks [5].

This paper exemplifies the communications that result from securing data that IoT devices hold using Blockchain-based Multi-mobile Code-driven trust Mechanism (BMCTM). Blockchain seems appropriate for tackling the security challenges in today’s Information and Communication Technologies (ICT) landscape with applications, like crypto-currency and anonymity. Since fog computing provides a distributed environment for computing, it demands distributed mechanisms to ensure secure data transactions across the networks, such as privacy and decentralized trust [17]. To this end, blockchain technology confers a decentralized security solution in fog-based IoT systems. For instance, when a new node joins the fog network, a consensus is run among fog nodes, or it may be run for the detection and isolation of compromised and malicious nodes from the network in a smart healthcare system. Therefore, blockchain supports fog computing by providing decentralized security solutions for an efficient and secured environment where centralized mechanisms may not meet the scalability and distributed nature of fog-based IoT systems [18]. We focus on why and how blockchain will positively impact IoT in saving energy, protecting devices, improving latency, and sustaining scalability. The fundamental concept of blockchain technology gives a basis for the cooperation between unknown and untrustworthy entities. It also supports the distributed nature of IoT, lacking a central security and authentication authority, as is the norm in current cloud computing architectures [16].

Hence, we demonstrate the benefits of blockchain in the context of internal attacks that IoT devices/sensors are subject to. We build-upon our previous work [9] to examine how to weave blockchain into a mobile-code, multi-edge system. In Reference [9], we proposed a software-defined-network-based Mobile Code-driven Trust Mechanism (MCTM), which was an energy-efficient solution for addressing internal attacks. Primarily, blockchain provides a distributed yet scalable trust information management, where the calculated trust values are recorded on the corresponding fog nodes. The mechanism proposed in this paper supports the distributed and scalable nature of IoT by following the temper-proof, decentralized, and immutable features of the blockchain. In addition, it also generates dynamic itineraries whenever a node joins or leaves the network to meet the dynamic nature of WSNs. The main contributions of this research are as follows:

1. A decentralized energy-efficient blockchain-based architecture for analyzing sensor networks’ behaviors to mitigate single-point-of-failure and scalability issues in centralized systems is proposed.
2. A novel yet reliable multi-mobile code-based mechanism is proposed for trust assessment that detects and isolates suspicious sensor networks to minimize network latency, traffic congestion, and message overhead for enhanced network lifetime.
3. Dynamic generation of itineraries are utilized to meet the dynamic nature of sensor node-powered IoT infrastructures for effective and in-time details gathering.
4. A proof-of-concept of trust assessment, along with some benchmark results, is presented.

The remainder of the paper is organized as follows: Section 2 presents the related work. The problem statement is discussed in Section 3. Section 4 provides the background of the technologies used in this framework. Trust architecture, assessment, calculation, and evaluation details are discussed in Section 5. Section 6 details the experimentation setup and experimental results. Finally, the conclusions are presented in Section 7, along with possible future directions.

## 2. Related Work

This section presents some existing research related to trust, blockchain, and mobile codes. To cater to blackhole and selective forwarding attacks, Airehrour et al. [19] proposed a trust mechanism that considered the number of packets delivered, along with the number of packets forwarded. However, it has more message overhead due to direct trust assessment and is not scalable in large deployments. In another work, Airehrour et al. [20] proposed a time-based trust-aware Routing Protocol for Low power and Lossy Networks (RPL) to cater to insider attacks in RPL-based IoT networks. However, the recommendation uncertainty was not considered, and the proposed mechanism was not energy efficient. Mehta and Parmar [21]  presented another trust-based tool for greyhole and wormhole attacks. An RPL-based trust routing mechanism was proposed by Khan et al. [22] for IoT devices to mitigate blackhole attack. It used a reputation-based approach for assessing the trust of IoT nodes. Besides, for clustered WSNs, Ozcelik et al. [23] proposed a hybrid Intrusion Detection System (IDS). Rafey et al. [24] proposed an adjustive distributed trust method to expand collaboration amongst the trusted nodes and adapt the trust values dynamically based on node conduct. However, this technique’s trust precision might be influenced by recommendations from false nodes that allot greater trust scores to their cluster of associates.

Distributed trust mechanisms have been proposed by Sicari et al. [25]. However, these procedures have been regarded as unbiased from transparency, accountability, and blockchain’s trust methods so far. Kang et al. [26] projected a reputation supported great-value information exchanging plan for vehicular systems utilizing an association of smart contracts, blockchain, and a subjective logic version, which depends on the transmission frequency, incident timelines, and trajectory resemblance for reputation administration. It proposed a distributed trust administration plan to compute the trustworthiness of switched over messages on the reputation scale of the spectator in blockchain supported vehicular systems. A Lightweight Scalable Blockchain (LSB) for IoT was proposed with an IoT affable agreement algorithm that includes a distributed trust mechanism to the block affirmation process [27]. Prevailing techniques for furnishing trust in blockchain-supported IoT applications either contemplate the data capture method for enhancing the trust in IoT information or the inter-node transmissions for all the nodes engaging in the blockchain system. As a result, they might not furnish end-to-end trust.

A blockchain-based privacy and data integrity framework was proposed by Satamraju [28] for IoT. Otte et al. [29] presented a decentralized and scalable trust mechanism (TrustChain) to mitigate Sybil attacks using blockchain to meet a myriad of IoT devices. Javaid et al. [30] proposed a scalable blockchain-based protocol for Internet of Vehicles (IoV) using smart contracts and a dynamic proof-of-work (dPoW) consensus algorithm. The proposed model ensures security by registering legitimate vehicles and isolating malicious ones. Another scalable blockchain-based trust model was presented as Blockchain Decentralized Interoperable Trust framework (DIT) [31]. They used smart contracts for authentication and validation using a private blockchain to ensure reliable communication in IoT-based healthcare systems. Similarly, Shala et al. [32] provided a comprehensive analysis of Machine-to-Machine Communication (M2M) applications, along with the trust and blockchain technology. They proposed an effective and scalable trust evaluation model for ensuring the trustworthiness of M2M peers. They stored data in the blockchain against data tampering attacks and ensured the detection of malicious peers [32].

A Near-Optimal Itinerary Design (NOID) algorithm is proposed by Gavalas et al. [33] that acclimatizes a process initially created for system structure concerns for the particular needs of WSNs. It identifies that mobile agents (MAs) accumulate information and may expand greatly during the visit of SNs. Hence, NOID restraints the number of visits made by every MA. Qi and Wang [34] proposed a couple of heuristic algorithms for MA itinerary planning using Local Closest First (LCF) and Global closest First (GCF) algorithms. These two algorithms are primarily linked to reduce calculative complications by Alsboui et al. [35]. Nevertheless, both LCF and GCF include the utilization of one MA that orderly calls on all SNs and do not calibrate accordingly when a single MA calls on a plethora of nodes. It shows that these algorithms are compatible with small-scale WSNs. Moreover, the dimensions of an MA extends uninterruptedly when the agent traverses between SNs. The expanding volume of the MAs out-turns in expanded utilization of the restricted wireless bandwidth and the consumption of the constrained power provisions of SNs.

Since its inception, mobile codes have gained attention in the industry and research. Its security is a concerning matter, as it visits a myriad of incognito and anonymous nodes [36]. In this regard, mobile agent security is fundamentally dependent upon mobile agent platform security to mitigate security threats [37]. The security of a mobile agent can be ensured using a variety of mechanisms. Karim [37] proposed the Tamper Proof Environments (TPEs), along with a time limit black-box security. They obfuscated code for the integrity of the data and its code. For the goodness and inspection of the mobile agents against malicious activities, Venkatesan et al. [38] proposed and enhanced a hybrid approach using the eXtended Root Canal Algorithm (XRCA) and Malicious Identification Police (MIP). Jolly and Batra [39] used Integrated Bloom Filter (IBF), along with the Ciphertext Policy Attribute-Based Encryption (CPABE) protocol, for the prevention of malicious code execution in a mobile agent.

## 3. Problem Statement

WSNs in the IoT paradigm have given rise to a new way of communication, denoted as Low power and Lossy Networks (LLN), where the resources of the SNs are very much constrained. Other limitations may include lossy links, low bandwidth, and communication affected by changes in the environmental conditions [9]. SNs are the backbone of WSN-based IoT infrastructures. Since sensory networks perform tasks without manual supervision and control (having constrained and limited resources), they further pose security challenges in SN-powered IoT applications. Therefore, adaptive security solutions are needed for such constrained infrastructures. Relying on existing security solutions, such as cryptography techniques, may successfully “stop” external attacks but miserably fail in front of internal attacks [17]. Apart from the security problems, another crucial element is the energy consumption in the lifespan of an SN [9]. Due to technical constraints, adopting traditional security methods is not feasible due to their high energy consumption [40,41,42]. Therefore, it is necessary to maintain a balance between energy depletion and proper security measures to ensure cost-effective and feasible solutions in the given context.

Another crucial factor that plays an important role in SN-powered IoT security is to strongly consider the physical deployment of such infrastructures, which has not been addressed widely so far. It is important to note that the deployment of SN-powered IoT infrastructures is arranged in a distributed fashion. However, the design of conventional IoT networks and its applications are deployed centrally to collect, store, process, and share data, ignoring these devices’ distributed constitution, which could also lead to certain security and privacy breaches [43]. The distributed and scattered deployments also require minimal delays in delay-sensitive applications, such as real-time data analysis in smart healthcare systems [16,44]. In this context, IoT applications create inevitable dilemma regarding proper security, energy consumption, network congestion, and latency, where latency issue may worsen with the substantial increase in the number of connected devices [45]. In addition, centrally managed security mechanisms may not ensure scalability in more distributed and largely prevalent SN deployments. Thus, a decentralized yet secure mechanism is also inevitable to deal with the distributively deployed SN-powered IoT infrastructures.

At present, IoT security approaches ignore their own computational complexity, data gathering, retrieval, transmission, and analysis [46,47]. This further increases the computational and memory overheads, which exhaust the resource-constrained devices. It also causes message overhead that leads to latency issues, making such mechanisms less efficient in current IoT deployment and an increased risk for malicious users [17]. Although several trust evaluation mechanisms focus on internal attacks, most of them are designed in a centralized-fashion where trust parameters are forwarded to a centralized entity in a multi-hop fashion, causing message overhead and energy depletion. Both message overhead and energy depletion play an important role in deteriorating network performance and lifetime [9]. Such mechanisms are only feasible when a small number of IoT devices are associated with the central entity. Nevertheless, in real life, SN-powered IoT devices are massive in number and deployed in a distributive-fashion. Thus, they overwhelm the central entity and the bandwidth resulting in latency. They are also highly susceptible to the single-point-of-failure  problem [16]. Besides this, the security of trust values and trustees themselves is very important. Therefore, considering all these issues, this research tends to propose a blockchain-based multi-mobile code-driven trust assessment mechanism.

## 4. Background

This section discusses blackhole and greyhole attacks (the focus of this study), blockchain, mobile codes, and itinerary planning.

### 4.1. Blackhole and Greyhole Attacks

The IoT networks, with their enormous scale, lack of sufficient storage, limited bandwidth capacity, and decentralized nature, renders them less stable and more susceptible to attacks. The attacks may be categorized as external and internal attacks. In an external attack, the adversary initiates an attack from outside the network (i.e., not a part of the system), whereas, if the adversary is a legitimate and authorized part of a network and initiates an attack, it is an internal attack. An adversary may drop data/control packets and deviate the routes in an internal attack. Examples of such attacks include blackhole and greyhole [48].

**Figure 1:**
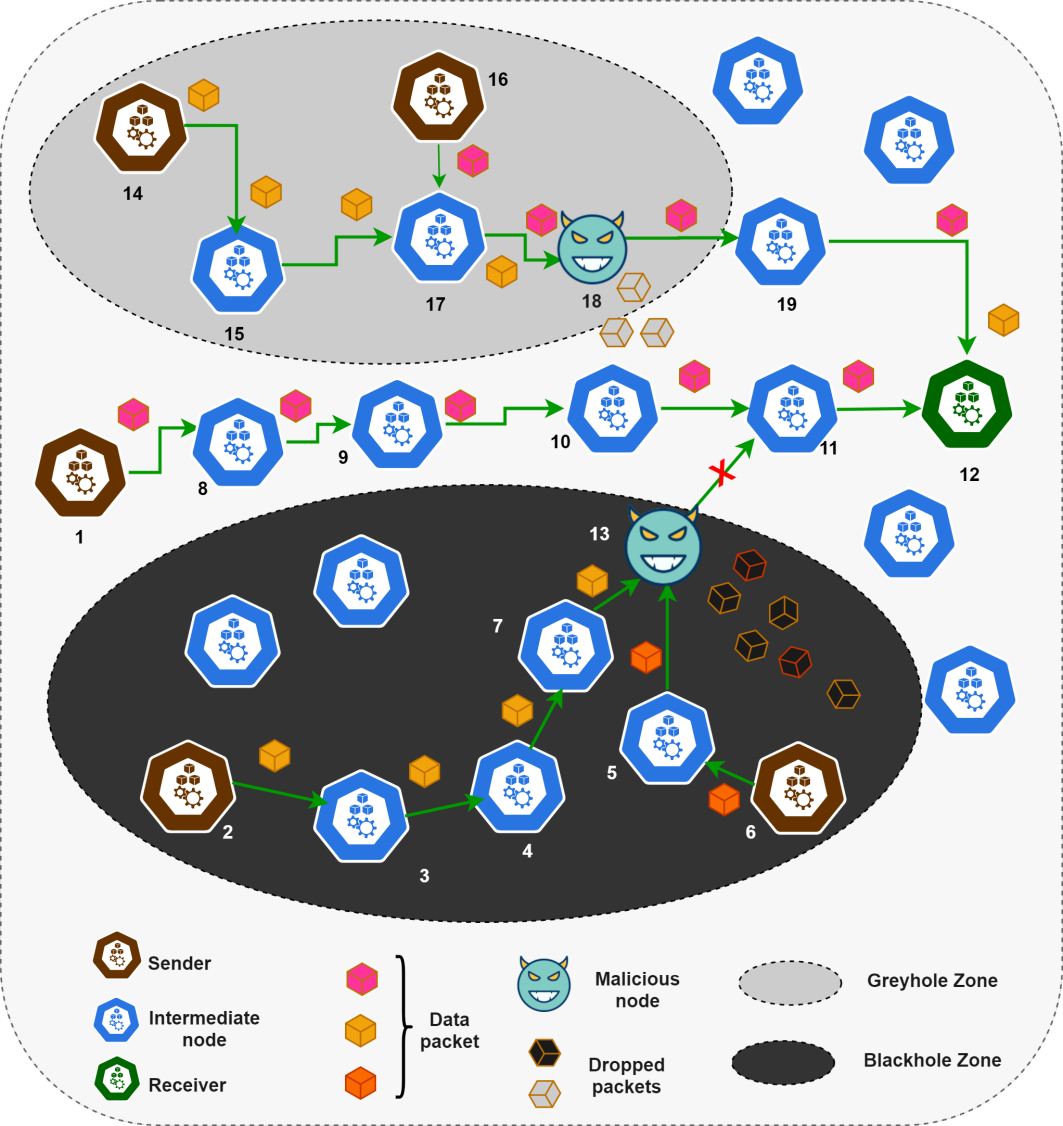
Blackhole and greyhole attack scenarios.

In a blackhole attack, all the receiving packets are dropped by the adversary node [9]. As illustrated in Figure 1, node 1, 2, and 6 relayed packets to node 12. The intermediate node 13 (a blackhole node) falsifies to be the nearest node to the destination (i.e., 12), generating a blackhole zone, for example. The nodes 2 and 6 route their packets via this node (node 13). The node 2 and 6 mistakenly choose the node 13 to be the optimal choice but, being malicious, node 13 will capture and drop all the packets received from 2 and 6. This degrades the performance and causes extensive power drainage due to re-sending of lost packet [9]. On the other hand, node 1 does not select this route, and all its packets are delivered to the destined node 12. Similarly, in a greyhole attack, only a few data packets are forwarded (from node 14 and 16 to node 12), and the rest are dropped cleverly to avoid detection [9] (as illustrated in the greyhole zone in Figure 1). The blackhole attack is easy to detect. It drops all the messages, whereas selective forwarding attacks are challenging, such as greyhole attacks. They circumvent detection covertly and intelligently by taking periodic packet loss, particular packet loss, and intermittent packet loss into account [49]. These kinds of attacks are a serious threat in large SN deployments, such as in IoT frameworks, where the sink node is far from the SN.

### 4.2. Blockchain Overview

Blockchain technology has become one of the most happening technologies in the 21st century due to its decentralized data storage mechanism in a peer-to-peer network. Satoshi Nakamoto introduced the blockchain concept as the supporting mechanism for the digital cryptocurrency called Bitcoin [50]. The technology develops a trusted environment by providing a single source of truth, a timestamped distributive ledger, eliminating a third party’s need. It discovers the trustworthiness of a certain transaction through irrefutable transaction evidence. It resolves the issue of double spending [51]. Interestingly, blockchain-based approaches present well-built counteractions to safeguard information while assisting the dispersed characteristics of the IoT. Because of the key features of blockchain, such as anonymity, decentralization, and security, it is a very useful technology to cater to the security and privacy problems in IoT systems in an easy, efficient, trustworthy, and secure manner [16,18,43].

**Figure 2:**
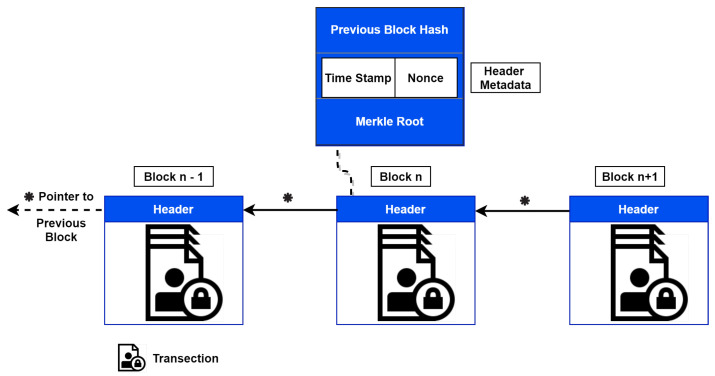
Composition of a block.

The blockchain is a (kind of) data structure with a chain of blocks where the connection between blocks is an address pointer based on a hash value [52]. All nodes independently hold their copy of the blockchain; the current “state” of the chain is calculated by processing each transaction to appear in the blockchain. Each block consists of six parts, as shown in Figure 2. It has a hash of the previous block, nonce (“number used once”), the hash of the current block, the Merkle root (hash of multiple transactions), a timestamp, and the transaction data [53]. Depending on the application, the block components may vary. The block headers contain metadata that helps block validation and links the previous blocks in the public ledger.

It uses consensus to add a new block in the chain. The majority of nodes in the network must agree on it. The smart contract protocol is invoked to validate a transaction in the blockchain [16]. After successful validation, the consensus protocol is initiated, which uses mining to add the transaction to a blockchain ledger. Mining is a process of validating new blocks in the chain. Once a transaction is validated, it is almost impossible to alter it: any retroactive alteration changes the hash of previous blocks resulting in breaching the consensus protocol. Therefore, the modified blockchain is rejected by the network. Every change in the ledger is recorded with a timestamp. All nodes/participants in the system possess identical copies of this ledger. After every validated transaction in the ledger, all participants update their copies. The consensus protocol addresses miners’ reward, mining time, mining process, signing transactions, selecting miners, and treating blockchain divergence.

There are different consensus protocols, such as Proof of Work (PoW) [17,51]. This protocol requires extensive computing power, time, and energy. Therefore, an alternative consensus protocol called Proof of Stake (PoS) [54] is proposed. Although the protocol is efficient, it also has some drawbacks, such as monopoly nodes holding high stakes. These nodes may manipulate or corrupt the system. To keep those powerful participants with a high stake in check, Delegated Proof-of-Stake (DPoS) [55] can be used. This consensus protocol reduces the issue of monopoly to some extent. However, some rich nodes can still come together to vote for these nodes that will act on their behalf in the wrong way. Since all these consensus protocols are computationally expansive, therefore, need for lightweight consensus protocol is inevitable. Ripple achieves consensus by using a Unique Node List (UNL) [56]. This protocol is energy efficient; however, it is more centralized somehow. It requires a vote from a limited number of selected nodes. Besides, IOTA [57] is another consensus mechanism, which is an energy- and time-efficient protocol. However, there is no facility to define smart contract rules governing the IOTA transaction compared to ethereum.

### 4.3. Mobile Code and Itinerary Planning

**Figure 3:**
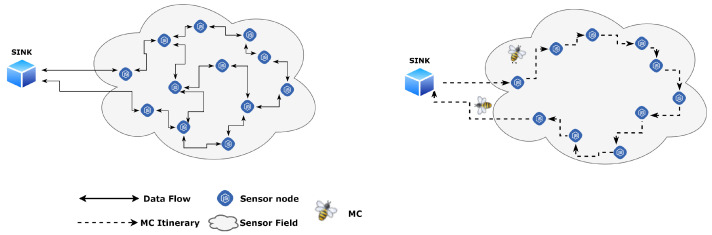
Data aggregation; multi-hop versus mobile code.

From the computer science perspective, trust evaluation can be described as the method of procuring a quantitative value for trust between at least two groups: a trustor and the trustee [58]. Trust-related credentials (also known as trust parameters or trust attributes) are gathered from all the participating SNs in an SN-powered IoT network to calculate trust. Unfortunately, the data aggregation in such networks requires significant improvement in low-powered low-resourced SN devices. Since data is forwarded in a multi-hop fashion, it not only generates unwanted traffic congestion but also depletes the energy of intermediate nodes, resulting in minimized network lifetime [59,60,61]. Therefore, the main objective is to minimize the network traffic, message overhead, and the power used in SNs, while aggregating information to enhance the network lifetime. As illustrated in Figure 3, with the help of the dissemination process via multi-hop communication, the sensory data moves to the sink, which is a remote processing element. To move this data, SNs rely on the available energy of the forwarding nodes. Moreover, neighboring SNs often generate redundant and correlated data. It makes a sharp necessity to develop methods that will eventually help decrease the amount of transmitted gathered data to the base station significantly [62]. Thus, one such process for aggregating data in WSNs involves a Mobile Code (MC), also known as a Mobile Agent (MA) [62]. It is harnessed in a distributed environment to overcome issues found in centralized environments, such as flexibility and scalability. It also conserves energy at the node level and enhances overall network lifetime and performance [59,60,61]. There could be several MAs crawling over many network clusters. However, the performance of an MA mainly depends upon the assigned plan provided by an itinerary. In addition, the size of a single mobile code may itself become a bottleneck and may contribute to latency and less scalability for a plethora of IoT devices [63]. Therefore, it is important to pay attention to the length of itineraries, such as long itineraries that may lead to traveling delays [64].

**Figure 4:**
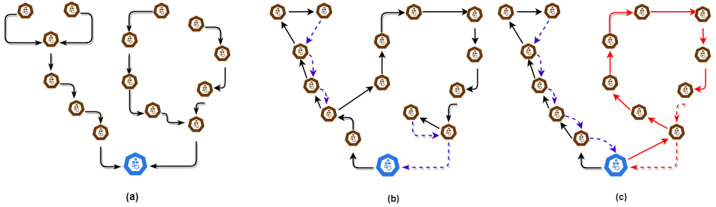
(**a**) Centralized data aggregation, (**b**) Single Itinerary Planning (SIP), and (**c**) Multiple Itinerary Planning (MIP).

The itinerary planning is divided into static, dynamic, and hybrid planning [65]. The static itineraries are computed before MCs’ migration, while dynamic itineraries work at every hop on the spot. In the hybrid ones, the sink or gateway decides the visiting SNs. The sequence of visits is determined by MC dynamically. Static itineraries work well than dynamic ones due to the pre-determined itineraries. They are more appropriate in monitoring, for instance, collecting data in physical quantities. They are further classified into Single Itinerary Planning (SIP) and Multiple Itinerary Planning (MIP). In the former, there is only one MC. In contrast, the latter has multiple MCs (as in our case) that traverse in parallel but for different clusters of IoT [65]. SIP has several setbacks: it is not scalable, and its performance degrades with the increase in the number of SNs. The large size of a network is the main hindrance in SIP because of traversal delay and energy depletion associated with data aggregation. In contrast, MIP elevates these issues. However, its design and execution are complex and challenging [66]. Figure 4 illustrates the phenomenon of centralized, SIP, and MIP data aggregation. The black arrows show a multi-hop data transition from different nodes to the sink. In contrast, blue and red arrows show MC itineraries.

## 5. Mobile Code-Enabled Trust Assessment

Before discussing the mechanisms for assessing trust in BMCTM, we first state our assumptions that the communication is secure, system admins are legitimate, and so are their actions.

### 5.1. Objectives

The increased numbers of sensing devices, connected as IoT networks, bring great digital interruption and chaos. These devices generate huge amounts of data sent either to a cloud or a fog server for further analysis and processing. The cloud-based infrastructures may fail to respond timely due to unwanted latency and delays, whereas fog is implemented in proximity to the IoT devices distributively to mitigate the latency issues [67,68]. The sensed data is forwarded in a multi-hop fashion. If any of the intermediate nodes gets compromised, projecting internal attacks, such as a blackhole or a greyhole attack, all or some of the data packets may be dropped, respectively. Therefore, due to the sensitivity of the situation, it is very important to ensure the constant flow of data in a trusted fashion. Trust as a security measure has been used extensively to mitigate internal attacks. However, conventional trust-based mechanisms are centralized and cannot meet the scalable nature of such infrastructures, and are highly prone to the single-point-of-failure problem. Besides that, blockchain technology provides a decentralized solution to meet scalability and mitigate single-point-of-failure issues [16]. In our case, the prime role of blockchain is to provide unforged, unaltered, and authentic trust values in a decentralized fashion.

The forged trust value may lead to adverse outcomes. For instance, in a smart healthcare scenario, if a malicious node is thought to be legitimate due to false trust value, the sensed data may never reach the destination in case of a blackhole attack, causing fatal consequences. Therefore, we need to ensure that the health specialists have received the sensed data to take appropriate and timely actions accordingly [69,70]. Our foremost aim is to provide a decentralized trust mechanism to mitigate the single-point-of-failure issue, which is the norm in centralized trust-based mechanisms. Secondly, we also aim at meeting the scalable nature of IoT infrastructures. Thus, blockchain is the best-suited platform with attributes, such as anonymity and encryption. It provides an immutable distributed ledger, where not only the value of trust is stored but also the devices‘ IDs, mobile codes‘ itineraries, and security-tokens are saved to ensure trustworthy data collection, which plays a vital role in an accurate and genuine trust assessment.

Keeping all the above in view, our objectives span 4 specific aspects that would allow us to assess trust in BMCTM.

1. Design and develop a reliable trust model to detect malicious nodes to enable trusted and reliable communication. This model should be efficient whether the situation is congested or sparse; the system must effectively monitor the trustworthiness of the underlying SNs. It must be robust and capable of resisting all the threats that may affect the trust assessment-related processes, such as detail gathering, trust calculation, and saving the calculated trust values. It should also be energy-efficient (i.e., minimizes overheads related to information collection, control, and computation). It must cast low message overhead to eliminate redundant transmission of messages by utilizing multi-mobile codes. It also improves network lifetime by eliminating frequent message exchange that generates excessive control traffic and quickly depletes intermediate node energy to improve network lifetime.
2. Have a decentralized approach to assess trust by producing accurate global trust, reducing network overhead, being scalable, managing resources efficiently, and being adaptable.
3. Improve scalability by ensuring that the proposed mechanism’s performance does not deteriorate if the number of connected devices (i.e., SNs) proliferates. For this reason, multi-MAs are initiated, as and when required, to meet the growing needs of a network.
4. Improve latency in 3 ways: by roving all the calculations to the corresponding fog nodes, which are close to the SNs (and not to the cloud or even not burdening SNs, which are already resource-constrained), by lowering network congestion that could play a vital role when SNs exchange trust-related information, and by improved detail-gathering to augment dynamic nature of WSNs using dynamic itinerary generation and initiation of MCs for effective and in-time trust assessment.

### 5.2. Building Blocks

BMCTM’s building blocks are detailed in the following:

- Infrastructure/Device layer: contains SNs deployed in different environments (later referred to as systems, such as healthcare systems and smart homes) for sensing, actuating, and communicating. The SNs belonging to different systems often need to communicate to exchange data for fulfilling the desired task. If any of the SNs becomes malicious, projecting either a blackhole or a greyhole attack, further communication may be abandoned (i.e., all messages get dropped in case of a blackhole- and some in case of a greyhole attack). In both the attack scenarios, the network performance and lifetime are affected badly. They may produce message and energy overheads on the victim and intermediate nodes due to re-sending (in case of multi-hop packet forwarding) of the lost packets. Therefore, to realize secure communications, the environment must be reliable and trustworthy. Hence, before data communication, an SN’s trust must be considered. The recipient will use the data in a good way instead of, for instance, modifying it, or misusing it. The proposed blockchain-enabled trust model prevents single-point-of-failure (as is the norm in centralized trust-based mechanisms), provides decentralized consensus about the addition and evaluation of trust, and scalability from meeting the decentralized yet exponentially growing IoT-based infrastructures. To ensure this confidence, we set 3 different thresholds, while considering the trustworthiness of an SN (discussed later in Section 5.3, Table 1).
- Fog layer: encompasses blockchain-enabled fog nodes supporting trust evaluation to establish and maintain a secure and reliable environment using different modules. They are provided by legitimate providers to perform the assigned tasks. Along with security (i.e., encryption), they also offer additional services, such as distributed device communication, storage, and processing [71]. The fog layer detects all connected devices, generates multiple itinerary plans, initiates multiple MCs according to the plans developed, calculates trust, and updates the blockchain at each transaction. The use of blockchain provides an immutable ledger for securing the current and historical data of trust values for all the concerned nodes. It ensures the integrity of trust values in trust-less environments, where data need to be secured against forgery and alteration. It also makes sure that the data aggregation (i.e., gathering trust parameters) is reliable by ensuring the integrity of MAs for evaluating error-free trust values that may be manipulated otherwise and result in disastrous outcomes. Each fog node consists of the following sub-modules and relies on some repositories:
  1. Device detector: detects the total number of SNs running in its respective cluster. To this end, the gateway provides the details of all connected devices, such as their IP address, MAC address, and type (sensor or actuator). It ships these details to the device list manager.
  2. Device list manager: receives the list of all legitimate SNs and their respective gateways from the device detector module. It maintains the details of the gateways and other connected devices. The details include locations, their coverage, and the systems they belong to. It also registers these SNs and their respective systems with the blockchain to further allow the system to deposit trust values against each device. Then, it sends them further to the dynamic itinerary plan generator to take further steps accordingly.
  3. Mobile code repository: keeps a security token list and generates multiple MCs according to the number of itinerary plans based on the number of SNs in a network cluster made by the dynamic itinerary plan generator module. There is an independent itinerary plan for each MC made by the dynamic itinerary plan generator module to visit the designated cluster.
  4. Dynamic itinerary plan generator: dynamically generates and initiates multiple MCs to traverse through the network. It will respond to whenever an SN joins or leaves the network and keep itself updated to generate new plans. These plans are then provided to the MC disseminator module, where MCs use them to traverse SNs for collecting SNs‘ forwarding behavior details and energy parameters. When an MC is initiated, a security token is also selected from the list and attached to it. The security tokens are mapped randomly on the initiation of each MC. A transaction is then made in the blockchain for the security token and the itinerary plan associated with each MC. They are cross-checked by the gateway on arrival to ensure their integrity.
  5. Mobile code disseminator module: lets MCs disseminate from the fog layer to the respective gateways. The MCs report back to it after trust-related data gathering. Together with the initiator module, it also maintains the history of visited or left gateways/SNs.
  6. Analyzer module: receives gathered data from the MC disseminator module and scrutinizes MCs on their return, along with device repository update. It is also responsible for trust assessment during scrutiny based on fetched details. The trust value is then transacted into the blockchain.

### 5.3. Assessment of Trust

This section encompasses details of different phases associated with information gathering, trust calculation, and malicious node detection and isolation.

- Setup phase: The authenticated fog nodes join in the blockchain network. These fog nodes make transactions into the blockchain to register systems and their corresponding nodes. Each fog node uses a unique system ID (IDentification) to register them in the blockchain network. The smart contract rules for a system registration are shown in Algorithm 1. It first checks for the system registration entry in the blockchain. Suppose the provided system registration does not exist in the blockchain; then, it checks for the gateway registration. If it also does not exist, the system is registered in the blockchain. After registering a system, its corresponding gateway is mapped to the system, and a mapping of the system and the corresponding fog node is performed. Similarly, the sensor nodes are also registered in the blockchain network using the public addresses assigned to these SNs. The algorithmic view of the smart contract rules for the registration of SNs is shown in Algorithm 2. It first checks for the system registration in the blockchain. If it exists, it is checked against the fog-system mapping. If it also exists, the sensor node registration entry is made in the blockchain. It then creates a system-node mapping and assigns an initial trust value as “50” to get into the system. We set the initial trust as 50 because if it is less than that, the smart contract will remove it from the system as if it is a malicious node.

It is important to note that the blockchain is implemented only on the fog nodes and on the gateways. In this view, there are three types of nodes in the proposed architecture, as shown in Table 2. The full node represents those fog nodes that mine transactions, validate blocks, and maintain the distributed ledger. The edge nodes represent the smart gateways. They do not make any transactions for state change (e.g., adding or altering data) in the blockchain. However, they can make a call function to read the needed values only. They save the block headers from mitigating data alteration or spoofing attacks [72], whereas the SNs refer to the smart devices deployed in the environment performing their assigned tasks.
**Algorithm 1.** System registration rules for smart contract
**Require:** *System Identification (sys.id), Gateway Identification (gt.id)*1:**Parameters:*****block_chain***: Blockchain ***System***: Object ***Gateway***: Object2:**if*** (system_exists(sys.id, block_chain) =* ***false**)* **then**3: **if** *(gateway_exists(gt.id, block_chain) =* ***false**)* **then**
4:  *register_SID(sys.id, block_chain)*
5:  *concerned_gateway(gt.id, sys.id)*
6:  *fog_system_mapping(sys.id, fog_address)*
7: **else**
8:  *return error()*
9: **end if**
10:**else**11: *return error()*
12:**end if****Algorithm 2.** Sensor node registration rules for smart contract
**Require:** *Node Identification (node.id)*1:**Parameters:*****block_chain***: Blockchain ***System***: Object ***Gateway***: Object2:**if***(System_exists(sys.id, block_chain) =**true**)***then**3: **if** *(fog_system_mapping(sys.id) = fog_address)* **then**
4:  *register_device(node.id, block_chain)*
5:  *system_node_mapping(sys.id, node.id)*
6:  *node_trust(node.id, “50”)*
7: **else**
8:  *return error())*
9: **end if**
10:**else**11: *return error())*
12:**end if**

- Information gathering phase: SNs maintain most current forwarding details of their neighboring SNs for trust evaluation. The forwarding behavior corresponds to the packets received, dropped, or forwarded from a particular node. Each node carefully observes and keeps a record of the successful and unsuccessful communication of neighboring nodes, along with their energy consumption (in our case). The MCs collect these data and ship them to the analyzer module for trust assessment. Before the detail collection, the MCs are verified by the gateway on their arrival. The gateway verifies the MC security token from the blockchain. If the security token does not match, they are considered tampered with or malicious and are not allowed to collect the trust details. Another MC is then sent as an alternative. Meanwhile, if a new SN(s) joins the network, its information is added to the gateway and a new itinerary plan is generated. It is important to note that MCs are generated according to the number of SNs in a cluster/network and not based on the number of clusters. Suppose new SNs further expand the corresponding system. In that case, the gateway updates its list and informs the fog node to update the list of attached SNs. In addition, another MC(s) is initiated for additional SNs, thus improving our proposed mechanism’s scalability. To assess the trust of newly joined-in SN(s), the gateway informs the fog layer. A new MC(s) (based on the number of the SNs that have joined in) is sent to collect the trust parameters on the fly.

**Figure 5:**
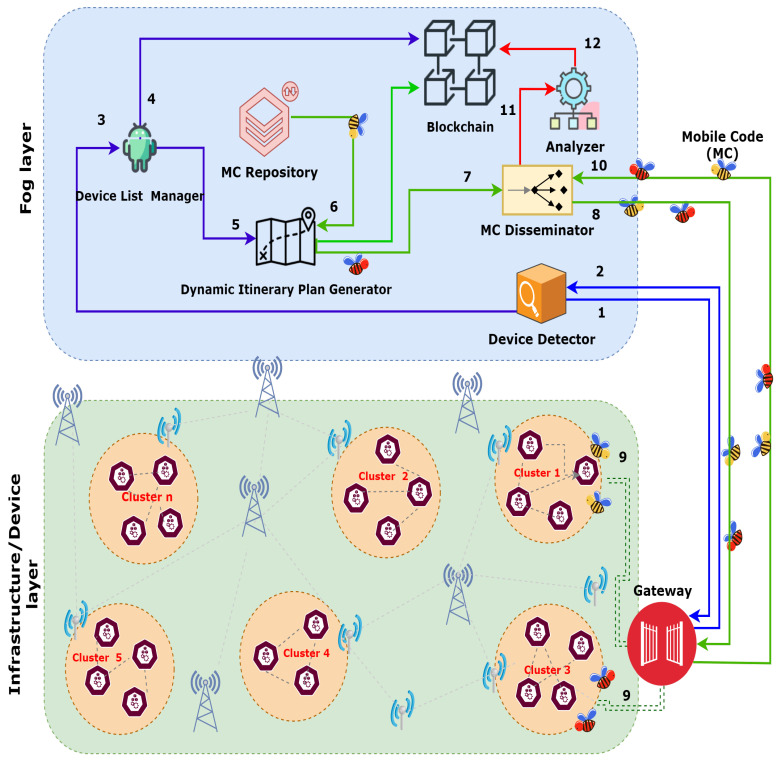
Process of collecting trust data from the Internet of Things (IoT) devices.

The whole process of data aggregation using multiple MCs and the dynamic itinerary generation is further pictured in Figure 5. The steps for the collection of trust data and trust evaluation are explained below:

1. The device detector module requests the gateway to discover the SNs of the corresponding system.
2. This information is sent from the gateway to the device detector module, which forwards the details to the device list manager.
3. The device list manager is updated, and step 4 is performed.
4. Upon receiving the SNs’ details, all the discovered nodes’ registration is made in the blockchain module.
5. The device list manager maintains the information of devices, along with their corresponding system. It sends the details to the dynamic itinerary plan generator module.
6. Depending upon the number of SNs, multiple MCs are initiated for each plan.
7. On its journey to the gateway, the intimated MC(s) further moves to the disseminator module.
8. The disseminator module sends the MCs to the designated gateway. The security token of each MC is evaluated on the gateway for validating the integrity of an MC.
9. If an MC is valid, it visits the designated SN cluster hop-by-hop and gathers the required details. After collecting details, it moves back to the gateway.
10. Then, it heads back to the fog layer via disseminator module.
11. The disseminator module sends these details to the analyzer module, which processes the obtained details, and the trust is assessed. The details of the trust calculation are further described in the proceeding section.
12. For each SN, the assessed trust value is further stored in the blockchain by making transactions to it, accordingly.

- Trust calculation phase: On the disposal of MCs, the gathered details are carried into the analyzer module for trust assessment. These details involve the information about forwarding behavior and energy consumption of each SN. Equation (1) (detailed in our previous work [9]) shows the mathematical way of calculating the remaining energy. If an SN is somehow compromised, the energy consumption will increase more than normal, resulting in the SN’s early evacuation.

To evaluate each smart device’s trust based on the gathered information, the analyzer uses the Subjective Logic Framework (SLF) [9]. There are three aspects of trust in SLF, which are: (1) Belief *b*, (2) Disbelief *d*, and (3) Uncertain *u*. The sum of these three attributes is always equal to 1. Forwarding behavior for a specific node can be recorded by estimating successful transaction *p* and unsuccessful transactions *n*. The three attributes of SLF can be calculated using Equations (2)–(4) given in Reference [9]. A constant value k is added to these values to avoid division by 0. Based on these three values, an IoT device’s final trust value is calculated by using Equations (5) and (6) (given in Reference [9]) and stored in the blockchain.

After collecting trust values, the next step is the insertion of the trust values into the blockchain. For a specific SN, the smart contract rules for adding or updating trust values in the blockchain are shown in Algorithm 3. The inputs in this algorithm are the trust value, node identification, system identification, and fog address.

According to the smart contract rules, once a fog node calculates the trust value successfully, it makes a transaction in the blockchain for storing the trust value. The transaction contains the SN identification and its trust value. The contract first checks for the system mapping with the requesting fog. If it exists, the fog node is verified. Suppose it is a valid fog node; then, the device registration is checked in the blockchain. The next step is to check the relationship between the devices and the system. If it exists, the trust value of the corresponding SN is updated in the blockchain, and mapping of trust and SN is created for future use. The main aim of making the restriction is to avoid the breach of the whole system security. For instance, if a smart home system’s fog node gets compromised, it should not affect other IoT devices’ trust values from another system. Another restriction is the insertion or alteration of trust value by another IoT device. The compromised node cannot edit or update the trust value of a trusted IoT device. It is done by verifying the signature of the fog node, making a transaction in the system.
**Algorithm 3.** Device trust insertion and update rules for smart contract
**Require:** Trust Value ($T_{v}$), Node Identification (node.id), System Identification (sys.id), Fog Address (fog_address)1:**Parameters:*****block_chain***: Blockchain ***System***: Object ***Gateway***: Object***Fog***: Object2:**if** (*fog_system_mapping(sys.id)* = *fog_address*) **then**3: **if** (*fog_verifier(fog_address,block_chain)* = *true*) **then**4:  **if** (*Node_exist(node.id)* = *true*) **then**5:   **if** (*system_node_mapping(sys.id)* = *node.id*) **then**6:    *update_trust(node.ID, $T_{v}$, block_chain)*
7:    *node_trust_mapping(node.id, $T_{v}$)*
8:   **else**
9:    return error()10:   **end if**
11:  **else**
12:   return error()13:  **end if**
14: **else**
15:  return error()16: **end if**
17:**else**18: return error()19:**end if**

- Malicious node detection and isolation phase: The final phase in the proposed mechanism is the malicious node detection and isolation phase. An SN from a network verifies the trust of another SN belonging to any system before communication begins. For instance, if node *A* wants to communicate with node *B*, it will initiate a direct transaction with fog layer blockchain. In response to that transaction, the fog layer provides the up-to-date trust value of node *B* to node *A*. Besides, blockchain is also deployed on gateways, as mentioned earlier. However, SNs can only initiate communication. In contrast, the thin data (i.e., block headers) is saved on the gateway blockchain. The consensus mechanism responsible for mining is deployed on the fog layer. The smart contract rules for the verification of trust values of the SNs are shown in Algorithm 4. It checks for the node and system mapping first and then checks for node existence in the blockchain. It then checks for the requesting gateway’s existence in the blockchain. If all are true, the trust value is returned.

**Algorithm 4.** Node trust verification rules for smart contract
**Require:** Node Identification (node.id), System Identification (sys.id), Gateway Identification (gt.id)1:**Parameters:*****block_chain***: Blockchain ***System***: Object ***Gateway***: Object***Fog***: Object2:**if** (*fog_system_mapping(sys.id)* = *fog_address*) **then**3: **if** (*Node_exist(node.id)* = *true*) **then**4:  **if** (*gateway_exist(gt.id)* = *true*) **then**5:   **if** (*concerned_gateway(gt.id)* = *sys.id*) **then**6:    return node_trust(node.id)7:   **else**
8:    return error()9:   **end if**
10:  **else**
11:   return error()12:  **end if**
13: **else**
14:  return error()15: **end if**
16:**else**17: return error()18:**end if**

Once the trust value is assessed, the smart contract will decide whether a node is trustworthy or not. In connection with the previous example, if node *B*’s trust value is above 75, it will be considered a highly trusted node. If the trust value is less than 50, the SN is considered not trusted and isolated or removed from the network. Algorithm 5 shows the smart contract rules for removing the malicious node from the network. This algorithm checks for the fog association to the system. It then confirms the system associated with the SN and confirms that the SN exists. If it is confirmed, the provided SN identification is removed from the network.

We have deployed three ratings for any node to assess its trustworthiness: highly trusted, moderately trusted, and malicious node. It is further illustrated in Table 1. If the data to be sent is highly confidential, it requires the SN to be highly trusted as well; otherwise, communication is not allowed by the smart contract. However, suppose the data is not much confidential. In that case, the moderately trusted node can also be allowed to take part in the communication.

Moreover, there is also an SN revocation mechanism implemented in the system. If the SN is identified as malicious, the corresponding fog node can remove it from the system. Only the corresponding fog node can do the removal of the SN and the update of data. It is for this reason that, if any of the fog nodes gets compromised, it cannot affect the trust values of any other SN in the system.
**Algorithm 5.** Node removal rules for smart contract
**Require:**  *Node Identification (node.id), System Identification (sys.id)*1:**Parameters:*****block_chain***: Blockchain ***System***: Object ***Gateway***: Object2:**if** (*fog_system_mapping(sys.id)* = *fog_address*) **then**3: **if** (*system_node_mapping(sys.id)* = *node.id*) **then**4:  **if** (*Node_exist(node.id)* = *true*) **then**5:   *remove_node(node.address,block_chain)*
6:  **else**
7:   return error()8:  **end if**
9: **else**
10:  return error()11: **end if**
12:**else**13: return error()14:**end if**


## 6. Experiments

**Figure 6:**
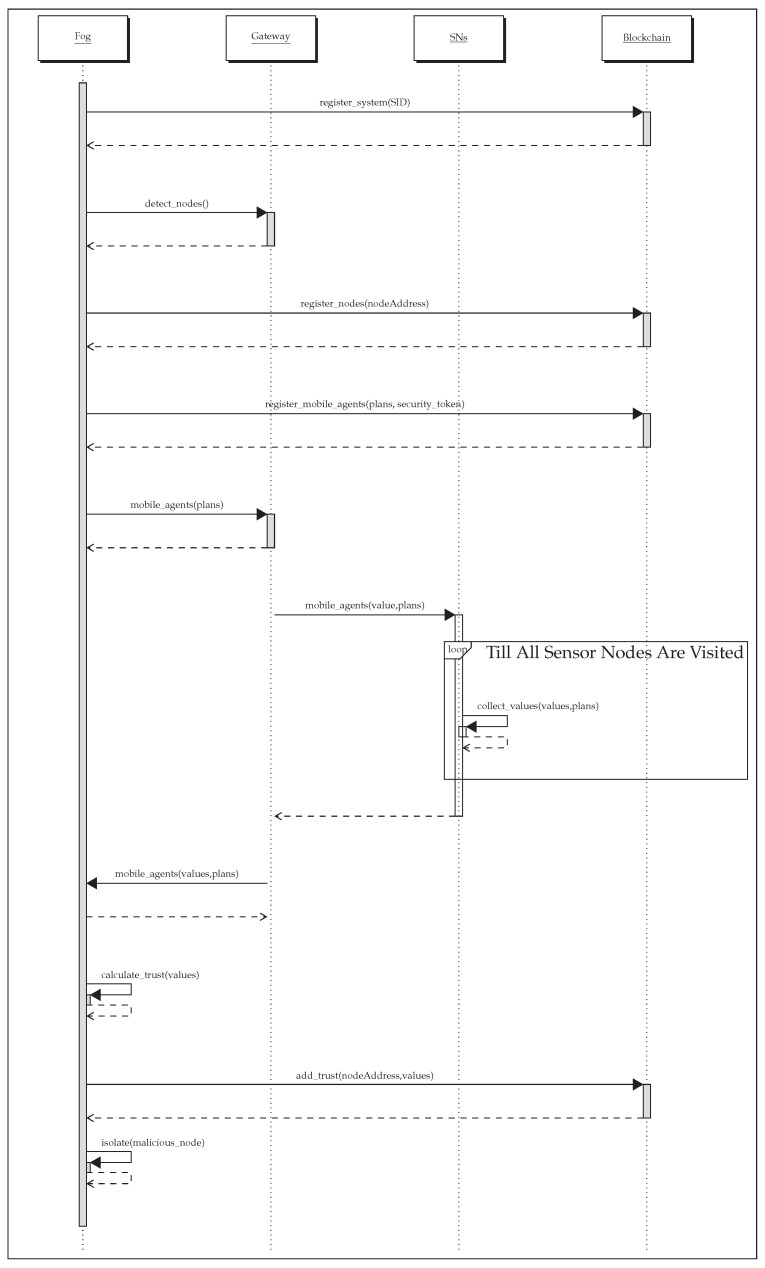
Sequence diagram of Blockchain-based Multi-mobile Code-driven trust Mechanism (BMCTM).

This section explains the experimentation setup and evaluation results concerning message overhead, malicious node detection time, and network lifetime. The details are given in the following subsections. The sequence diagram in Figure 6 presents the overview of the steps involved in the trust calculation and isolation of malicious nodes.

### 6.1. Experimentation Setup

For diagnostic and testing, simulation is considered to be a fundamental need. No doubt, it has become a standard in the domain of networking and WSNs. However, it might be insufficient to address the real-world deployments [73]. Thus, it is crucial to design real experimentation to address the simulation gaps. Secondly, there is no blockchain-based simulator available currently (to the best of our knowledge) that fulfills our needs and provides resulting values corresponding to the proposed parameters. Therefore, we prefer a test-bed experiment that provides a functional understanding of the underlying systems. It may also help observe certain behaviors and conditions that might get skipped during the simulation. In the experiments, we use five nodes in total, out of which one is the fog node, one acts as the actuator, and the rest are normal SNs. Initially, there is no malicious node in the network. We then introduce one malicious node in the network and observe the functionality. The fog node generates two itinerary plans and initiates 2 MCs for each plan, accordingly. Five different iterations are used for the blackhole and greyhole attack scenarios in both BMCTM and MCTM [9] approaches. In each iteration, we randomly make any node malicious. The reason behind these iterations is to verify the consistency of the proposed mechanism. We note the effects and the functionality of both the mechanisms in each iteration. We take an average value of all the five iterations to represent the results discussed in later sections.

This section provides particulars of different tools (also listed in Table 3), environments, and experimentation setup details. For the implementation of Ethereum, we use the Ganache and BLOCKBENCH emulator. The Truffle suite and Remix emulators are used to deploy smart contracts with a solidity compiler to compile smart contracts. Furthermore, to provide an Integrated Development Environment (IDE) interface, Atom is used for developing nodes. We use two environments in this experimentation. JavaScript (nodeJS) and solidity are used to realize nodes and develop smart contracts, respectively. The nodes are made in nodeJS, a user-friendly and emerging platform for IoT application development [74]. The message passing is done using Transmission Control Protocol (TCP) connection, as it confirms the message delivery and is recommended in IoT scenarios [75]. That is why we prefer TCP over User Datagram Protocol (UDP), as it makes sure whether the message has been delivered or not. The fog node initiates the MCs, fetches each node’s forwarding behavior and remaining energy, and brings the data back to the fog node. This data is shipped to the analyzer module, where the trust value is calculated and sent to the blockchain. The trust value is later used whenever an SN starts communication to determine the desired node’s trustworthiness.

### 6.2. Results

This section details the results drawn from the experimentation.

#### 6.2.1. Calculating Message Overhead

**Figure 7:**
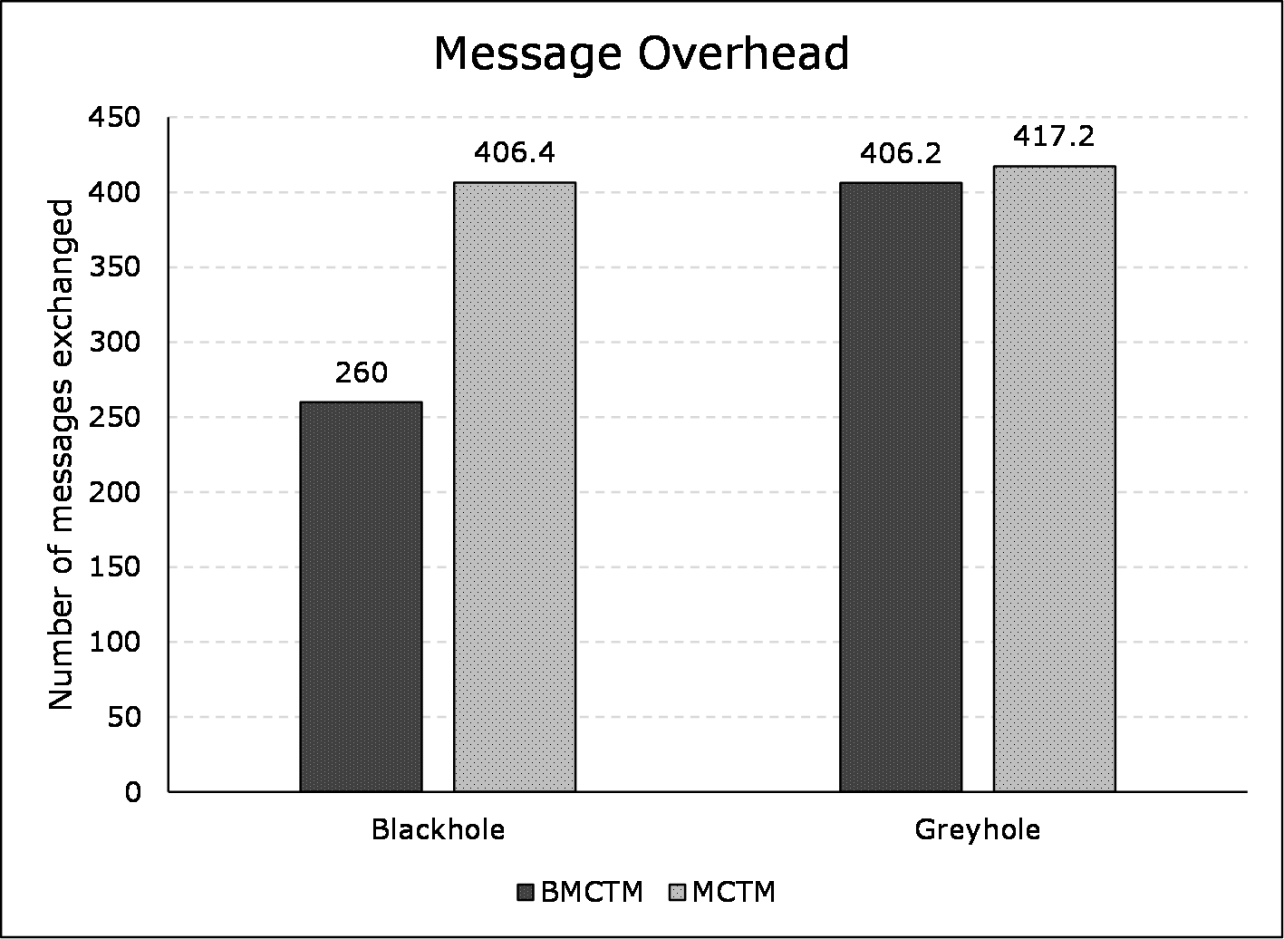
Message overhead in BMCTM and Mobile Code-driven Trust Mechanism (MCTM) in blackhole and greyhole attack scenarios.

The message exchange ratio determines the message overhead as an exchange of messages, in a network, between 2 nodes. Figure 7 represents the average message overhead ratios of BMCTM and MCTM, respectively. It illustrates the message overhead in BMCTM and MCTM in blackhole attack scenarios, which shows a 43.94% improvement (i.e., less message overhead in BMCTM), whereas in case of BMCTM and MCTM in a greyhole attack scenario has 2.67% less message overhead for the BMCTM scheme. It depicts fewer message exchanges in BMCTM than MCTM for both the blackhole and the greyhole attacks. The results reveal that the multi-mobile code-based mechanism is better than the single mobile code-based mechanism. It is because multi-mobile codes fetch the details of their assigned itineraries. It benefits in lower message exchange, less network traffic congestion, and smaller sized MC.

#### 6.2.2. Malicious Node Detection Time

**Figure 8:**
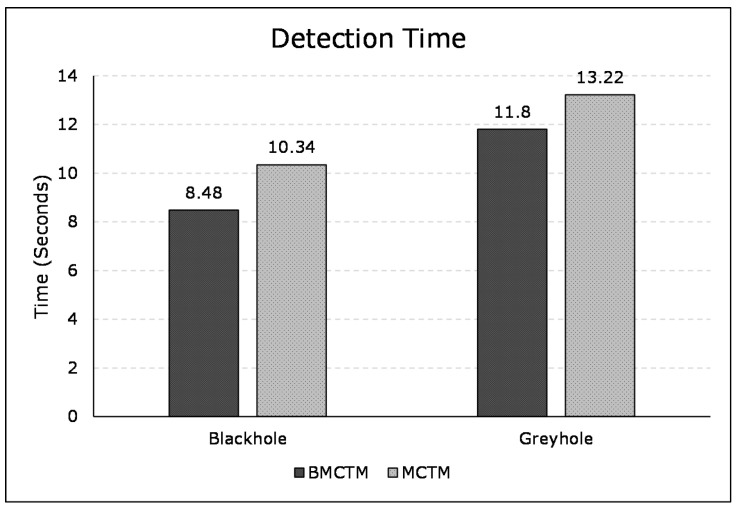
Malicious node detection time of BMCTM and MCTM in blackhole and greyhole attack scenarios.

Detection time is referred to as the time a mechanism takes to detect and isolate the malicious SNs from a network. Figure 8 illustrates the detection time of blackhole and greyhole attacks for both BMCTM and MCTM schemes, respectively. It represents that the time to detect blackhole attack is 20.35% less in BMCTM than in MCTM. Similarly, it shows 11.35% improvement in detection time (i.e., lower) for BMCTM compared to MCTM. In the same fashion, it also illustrates the detection time difference of blackhole and greyhole attacks in BMCTM and MCTM. The figure shows that the time taken to detect a blackhole attack is 8.48 s and 10.34 s in both BMCTM and MCTM, respectively. In contrast, it is 11.8 s and 13.22 s for greyhole detection, respectively. This difference shows that the detection of blackhole attacks takes relatively less amount of time than the detection of greyhole attacks. This is because, in blackhole attack scenarios, all packets are dropped. Hence, it is easier to detect such malicious nodes than those in greyhole attack, where packets are dropped less frequently.

#### 6.2.3. Network Lifetime and Energy Model

**Figure 9:**
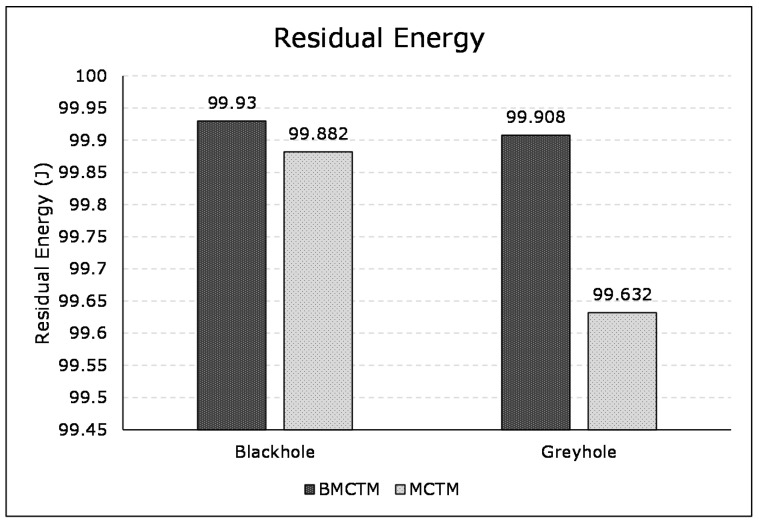
Residual energy of BMCTM and MCTM in blackhole and greyhole attack scenarios.

An SN is considered dead if its energy drains fully. Simultaneously, a network’s lifetime depends on the first node’s collapse upon its complete depletion of energy. Figure 9 demonstrates the average residual energy of the network in both the greyhole and blackhole attack scenarios. It shows that, while detecting and isolating of blackhole attack, the average residual energy of BMCTM is 99.93 J, while it is 99.882 J for MCTM. This shows 0.048% more residual energy in BMCTM than in MCTM. Similarly, the residual energy of BMCTM is 99.908J and that of the MCTM scheme is 99.632J, with a difference of 0.045% in the greyhole attack scenario. We followed the same initial energy parameters and energy consumption model in both scenarios for the two schemes to remain in line with the same environment as was proposed in Reference [9], as shown in Table 4. The BMCTM established better results due to relatively less message overhead, multi-mobile codes, and no trust-related computations at the node level.

#### 6.2.4. Throughput

**Figure 10:**
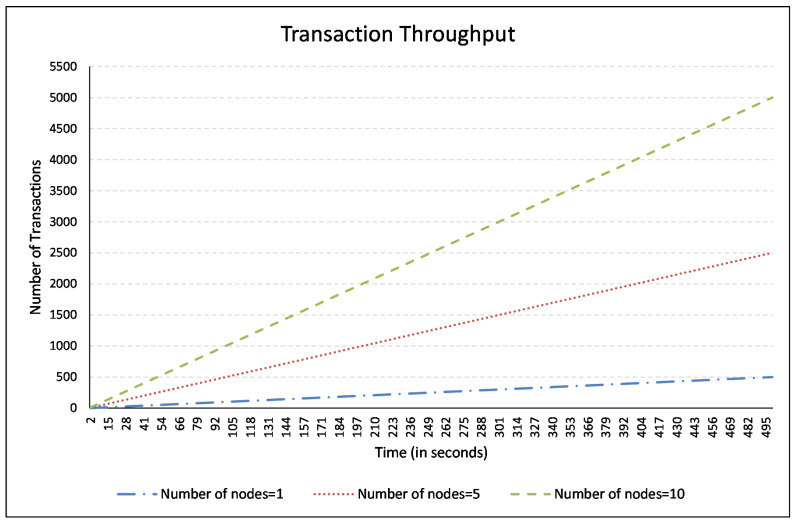
Transaction throughput in BMCTM.

**Figure 11:**
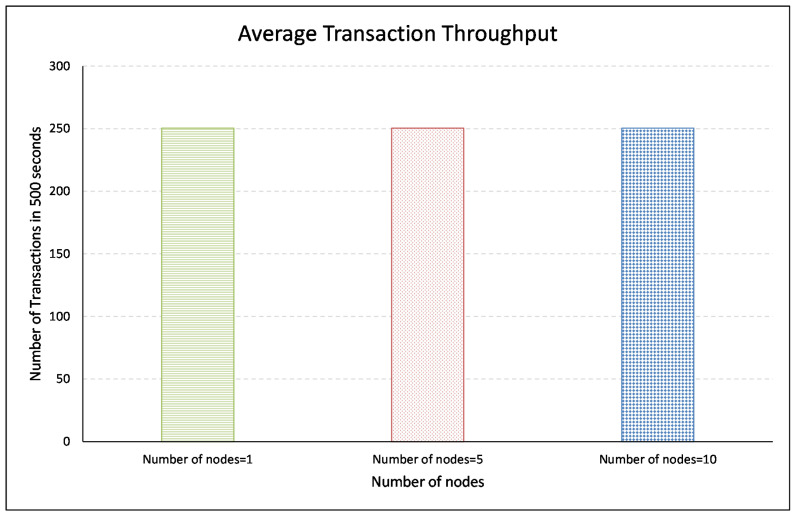
Average transaction throughput in BMCTM.

The throughput (in our case) is referred to as the degree of transactions recording in the blockchain in a scalable and efficient way. A mechanism is “scalable” if it does not deteriorate with an increasing number of connected nodes and works invariably. To validate the proposed mechanism’s scalability, we ran the mechanism for 1, 5, and 10 nodes and compared the throughput. Figure 10 illustrates that one node transacted 500 transactions into the blockchain in 500 s, 5 nodes made 2500, and 10 nodes made 5000 transactions. It may be noted that for an increased number of transactions, the proposed mechanism took the same time (i.e., 1 s). It is further elaborated in Figure 11, which represents an average transaction throughput of BMCTM. It shows an average number of transactions made in 500 s for 1, 5, and 10 nodes. On average, one node made 250 transactions. Similarly, on average, 5 and 10 nodes also made the same number of transactions in the same amount of time, which shows that the proposed mechanism remained persistent. It did not deteriorate with an increasing number of nodes and took the same time as was taken by one node only.

Hence, the results, discussed in Section 6.2 uphold the reduced message overhead, more residual energy, scalability of the proposed model in terms of throughput, and an optimized lifetime of the underlying network using BMCTM under greyhole and blackhole attacks with the same setup environment, parameters, and energy model.

## 7. Conclusions

Generally, WSNs are used to accumulate and disperse information. Hence, while gathering information, one should choose only trusted information resources and data. Since the SNs are the data sources, a sensor might be seized by an adversary or an SN itself might turn unreliable. Therefore, trust mechanisms are a crucial prerequisite for mitigating such attacks. Designing a robust trust-based security framework needs to meet several concerns, such as the diverse, mobile, scalable, distributed, and restrained nature of SNs. Existing centralized trust assessment mechanisms may face the single-point-of-failure problem, as well as scalability, traffic congestion, computation, communication, and message overheads. These overheads deplete an SN’s energy, which consequently threatens the lifespan of the whole network. Thus, this paper presents an energy-efficient blockchain-based multi-mobile code-driven trust mechanism for detecting internal attacks in sensor node-powered IoT. Dynamic itineraries are generated for multiple mobile codes depending upon the number of SNs to reduce traffic congestion problem and message overhead. The gathered details are brought into the fog nodes. Trust is calculated and transacted into the blockchain to support the distributed nature of SN deployment, provide scalability, and avoid the single-point-of-failure problem. The results demonstrated improvements in mitigating message overhead, improved malicious node detection time, scalability, and optimized network lifetime and performance. A further extension would include identifying fog nodes and authentication of admins while securing the communication between two nodes using blockchain technology. In support of this, we intend to implement and test the proposed blockchain-based mechanism using a real-life scenario, such as smart healthcare.

## Figures and Tables

**Table 1:** Classes of device trust.

Trust Type	Trust Value Range
Highly Trusted	75 ≤ Trust Value ≤ 100 && Residual Energy ≥ 50
Moderately Trusted	50 ≤ Trust Value ≤ 74 && Residual Energy ≥ 50
Malicious	Trust Value ≤ 49 && Residual Energy < 50

**Table 2:** Types of nodes in proposed system architecture.

Node Type	Representative Node Role(s)
**Full Node**	- Fog Servers
	- Generate itinerary plans
	- Initiate and validate mobile codes
	- Assess trust values
	- Perform mining
	- Validate blocks
	- Store and maintain distributed ledger
	- Isolate malicious nodes
**Edge Node**	- Smart Gateways
	- Store only the block headers
	- Verify mobile codes
**Thin Node**	- Smart Device
	- Performs only assigned task

**Table 3:** Tools used in the experimentation.

Tool	Usage
Ganache	Ethereum emulator.
BLOCKBENCH	Ethereum emulator.
Truffle Suite	For the compilation and deployment of the smart contract.
Solidity	For the development of Smart Contract.
JsonRPC	For the realization of communication between node and blockchain.
Remix	IDE for developing smart contract.
Red NodeJS	For developing SNs.

**Table 4:** Experimentation parameters.

Environment	Details	
	Parameter	Value
	Gateway	1
	Fog Node	1
	Number of Nodes	4
Energy Consumption Model	Node Initial Energy	100 J
	Standby Power	0.708 mJ/s
	$E_{rx}$	0.0009 mJ/bit
	$E_{tx}$	0.0010875 mJ/bit
